# Phellinus igniarius-derived carbon dots suppress liver cancer through the ROS/MAPK signaling axis

**DOI:** 10.3389/fphar.2026.1915586

**Published:** 2026-09-09

**Authors:** Yinan Li, Mengyao Wu, Xiuhe Zhao, Jie Zhang

**Affiliations:** 1 Department of Pharmacy, Tianjin Medical University Cancer Institute and Hospital, National Clinical Research Center for Cancer, Tianjin’s Clinical Research Center for Cancer, Key Laboratory of Cancer Prevention and Therapy, Tianjin, China; 2 Tianjin Key Laboratory of Technologies Enabling Development of Clinical Therapeutics and Diagnostics, School of Pharmacy, Tianjin Medical University, Tianjin, China

**Keywords:** carbon dots, liver cancer, MAPK signaling, Phellinus igniarius, reactive oxygen species

## Abstract

Liver cancer is one of the most common and highly lethal malignancies worldwide, and current therapeutic strategies remain limited, posing significant clinical challenges. Thermal processing of natural products is not only used for the extraction of active constituents, but may also induce structural reconstruction and bioactivity transformation of endogenous components. Recent studies have demonstrated that certain natural products can generate carbon dots (CDs) with specific biological activities under high-temperature conditions, suggesting that these nanoparticles may serve as novel active components formed during thermal processing. Phellinus igniarius, a traditional medicinal fungus, possesses anti-inflammatory and immunomodulatory activities. However, the formation of carbon dots during its thermal processing and their biological functions have not been systematically investigated. In this study, Phellinus igniarius-derived carbon dots (PI-CDs) were synthesized using a hydrothermal method, and their antitumor activities and underlying mechanisms were systematically evaluated both *in vitro* and *in vivo*. The results showed that PI-CDs exhibited uniform nanoscale morphology with good dispersibility. *In vitro* experiments demonstrated that PI-CDs significantly inhibited the viability, migration, and invasion of liver cancer cells in a concentration-dependent manner. Transcriptomic analysis revealed significant enrichment of MAPK signaling and apoptosis-related pathways, which was further supported by increased phosphorylation of p38 and JNK. Mechanistically, PI-CDs induced intracellular reactive oxygen species (ROS) accumulation, promoted mitochondrial membrane depolarization, and triggered apoptotic cell death. Furthermore, PI-CDs effectively suppressed tumor growth *in vivo* xenograft model without obvious systemic toxicity. Collectively, these findings suggest that PI-CDs exert antitumor effects through oxidative stress-associated MAPK signaling and mitochondria-dependent apoptosis. This study demonstrates that carbon dots generated from the medicinal fungus Phellinus igniarius possess significant anti-liver cancer activity and reveals a ROS/MAPK-associated mechanism underlying their biological effects. More importantly, our findings highlight thermal processing as a promising strategy for transforming traditional medicinal resources into functional nanomaterials with intrinsic therapeutic potential, thereby providing a new perspective for the development of natural product-derived nanomedicines for cancer treatment.

## Introduction

Liver cancer is one of the most prevalent and lethal malignancies worldwide, representing a major global health burden with high incidence and mortality rates (Sung et al., 2021). The disease burden of liver cancer is particularly severe due to its high invasiveness, frequent recurrence, and poor prognosis (Han et al., 2024; Cao et al., 2021). Owing to its insidious onset and strong metastatic potential, most patients are diagnosed at advanced stages, which greatly limits therapeutic efficacy (Li et al., 2024). Although current treatment strategies, including surgical resection, chemotherapy, targeted therapy, and immunotherapy, have improved clinical outcomes to some extent, challenges such as drug resistance, systemic toxicity, and postoperative recurrence remain unresolved (Anwanwan et al., 2020; Xue et al., 2024). Therefore, the development of safe and effective therapeutic approaches for liver cancer remains urgently needed.

Carbon dots (CDs), as a class of emerging carbon-based nanomaterials, have attracted increasing attention because of their ultrasmall size, excellent water solubility, favorable biocompatibility, stable photoluminescence, and tunable surface functionalization (Liu et al., 2025; Singh et al., 2025). In addition to their applications in bioimaging and drug delivery, accumulating evidence suggests that CDs synthesized from specific precursors may exhibit intrinsic antitumor activities (Zhang et al., 2024; Shen et al., 2022). Notably, natural products and traditional Chinese medicines have recently been explored as promising carbon precursors for CD synthesis. Compared with conventional synthetic precursors, naturally derived CDs often possess unique physicochemical characteristics and biological activities owing to the complex composition of their source materials (Yan et al., 2025). Recent studies have demonstrated that carbon dots can be generated from various natural resources, including medicinal plants, herbs, and other bioactive materials, through hydrothermal or thermal carbonization processes. These naturally derived CDs have been reported to retain or acquire biological activities, such as antioxidant, antibacterial, and antitumor effects, suggesting that thermal conversion of natural products may represent a potential strategy for generating functional nanomaterials (Aytar et al., 2025; Gedda et al., 2023; Zheng et al., 2026). These findings provide new opportunities for the development of multifunctional nanobiomaterials with potential biomedical applications.

Phellinus igniarius (PI) is a traditional medicinal fungus widely used in East Asian medicine, has been reported to possess anti-inflammatory, antioxidant, immunomodulatory, and antitumor activities (Wang et al., 2018; Wang et al., 2019). Previous studies have mainly focused on its polysaccharides, flavonoids, and phenolic compounds, which are considered the major bioactive constituents responsible for its pharmacological effects (Zhu et al., 2023). However, the chemical transformations and potential generation of novel bioactive nanostructures during thermal processing of PI remain largely unexplored. In particular, whether thermally processed PI can generate bioactive CDs with antitumor properties has not yet been systematically investigated.

Reactive oxygen species (ROS)-mediated oxidative stress has been recognized as an important mechanism underlying nanoparticle-induced tumor cell death. Excessive ROS accumulation can disrupt intracellular redox homeostasis, induce mitochondrial dysfunction, and ultimately trigger apoptosis. Recent studies have demonstrated that certain natural product-derived CDs are capable of regulating intracellular ROS levels and promoting tumor cell apoptosis through oxidative stress-related pathways. These findings suggest that naturally derived CDs may serve as promising nanotherapeutic agents for cancer treatment.

In the present study, Phellinus igniarius-derived carbon dots (PI-CDs) were synthesized via a hydrothermal method, and their antitumor activities and underlying mechanisms were systematically evaluated both *in vitro* and *in vivo*. We further investigated whether PI-CDs exert antitumor effects through ROS accumulation, disruption of redox homeostasis, and induction of mitochondria-dependent apoptosis. This study provides experimental evidence for the development of medicinal fungus-derived nanomaterials and offers new insights into bioactive nanocomponents generated during thermal processing of natural products.

## Materials and methods

### Preparation and characterization of PI-CDs

Phellinus igniarius-derived carbon dots (PI-CDs) were synthesized via a hydrothermal method. Briefly, 1.5 g of Phellinus igniarius powder was dispersed in 30 mL ultrapure water under ultrasonication, followed by transfer into a Teflon-lined autoclave and heated at 180 °C for 10 h. After cooling to room temperature, the reaction mixture was filtered through a 0.22 μm membrane, and the filtrate was dialyzed using a 500 Da dialysis membrane against ultrapure water for 24 h. The dialyzed solution was then lyophilized to obtain PI-CDs powder, which was subsequently redispersed in ultrapure water for further experiments. The morphology and size distribution were characterized using transmission electron microscopy (FEI, Czech Republic). Hydrodynamic size was analyzed using a dynamic light scattering system (Malvern, UK). Surface functional groups were determined by Fourier transform infrared spectroscopy (Thermo Fisher Scientific, USA) using the KBr pellet method. UV–vis absorption spectra were recorded using a UV–vis spectrophotometer (Thermo Fisher Scientific, USA), and fluorescence spectra were obtained using a fluorescence spectrophotometer (Edinburgh Instruments, UK).

### Cell culture

Human hepatocellular carcinoma HepG2 cells, human breast cancer MCF-7 cells, and human lung cancer A549 cells were obtained from Jiangsu KeyGEN BioTECH. HepG2 cells were cultured in MEM medium, while MCF-7 and A549 cells were maintained in DMEM medium, both supplemented with 10% fetal bovine serum and 1% penicillin-streptomycin. Cells were cultured in a humidified incubator (Thermo Fisher Scientific, USA) at 37 °C with 5% CO_2_ and passaged every 2–3 days. Cells in logarithmic growth phase were used for all experiments.

### Cell viability assay

Cell viability was assessed using a CCK-8 assay kit (Absin, China). Cells were seeded into 96-well plates at a density of 5 × 10^3^ cells per well and incubated for 24 h. HepG2 cells were then treated with different concentrations of PI-CDs for 48 h. Subsequently, CCK-8 solution was added and incubated for 1–2 h at 37 °C. Absorbance was measured at 450 nm using a microplate reader (Bio-Rad, USA), and cell viability was calculated accordingly.

### Transwell migration and invasion assays

Cell migration and invasion were evaluated using Transwell chambers (Corning, USA). For migration assays, 2 × 10^4^ HepG2 cells suspended in serum-free medium were seeded into the upper chamber, while medium containing 10% FBS was added to the lower chamber as a chemoattractant in the presence or absence of PI-CDs. For invasion assays, the upper chamber was pre-coated with Matrigel (Corning, USA) and incubated at 37 °C for 30 min prior to cell seeding. After 24 h incubation, non-migrated or non-invaded cells were removed, and the remaining cells were fixed with 4% paraformaldehyde and stained with 0.1% crystal violet. Cells were visualized and quantified under an inverted microscope (Olympus, Japan).

### Intracellular ROS detection

Intracellular ROS levels were measured using a DCFH-DA ROS assay kit (Solarbio, China). HepG2 cells were treated with PI-CDs (30, 60, and 120 μg/mL) for 24 h, washed with PBS, and incubated with DCFH-DA at 37 °C for 30 min in the dark. Fluorescence intensity was detected using a flow cytometer (BD Biosciences, USA).

### Mitochondrial membrane potential assay

Mitochondrial membrane potential was assessed using a JC-1 assay kit (Solarbio, China). After treatment with PI-CDs for 24 h, cells were incubated with JC-1 working solution at 37 °C for 20 min in the dark, washed with JC-1 buffer, and analyzed by flow cytometry (BD Biosciences, USA) according to the manufacturer’s instructions.

### Apoptosis analysis

Cell apoptosis was detected using an Annexin V-FITC/PI apoptosis detection kit (Beyotime, China). After PI-CDs treatment for 24 h, cells were harvested, washed, and resuspended in binding buffer. Cells were incubated with Annexin V-FITC for 5 min in the dark, followed by PI staining. Samples were analyzed using a flow cytometer (BD Biosciences, USA).

### Animal experiments

Male BALB/c nude mice (4–6 weeks) were purchased from Vital River Laboratory Animal Technology Co., Ltd. HepG2 cells were resuspended at 5 × 10^7^ cells/mL, and approximately 5 × 10^6^ cells were subcutaneously injected into each mouse to establish xenograft tumor models. When tumor volume reached approximately 80–100 mm^3^, mice were randomly divided into groups and treated with PI-CDs (10, 20, and 40 mg/kg) via intratumoral injection every 3 days for 3 weeks. Tumor size was measured using a digital caliper every 3 days, and tumor volume was calculated using the formula: V = 0.5 × L × W^2^. Body weight was recorded throughout the experiment. At the end of the study, mice were euthanized by cervical dislocation, and tumor tissues were excised and weighed. All animal experimental procedures were approved by the Animal Ethics Committee of Tianjin Medical University (No. TMUaMEC202627) and were conducted in accordance with institutional guidelines for the care and use of laboratory animals.

### Statistical analysis

All data are presented as Mean ± SD. Statistical analysis was performed using GraphPad Prism software. Student’s t-test was used for comparisons between two groups, and one-way analysis of variance (ANOVA) was used for multiple group comparisons. A value of p < 0.05 was considered statistically significant.

## Results

### Characterization of Phellinus igniarius-derived carbon dots

**FIGURE 1:**
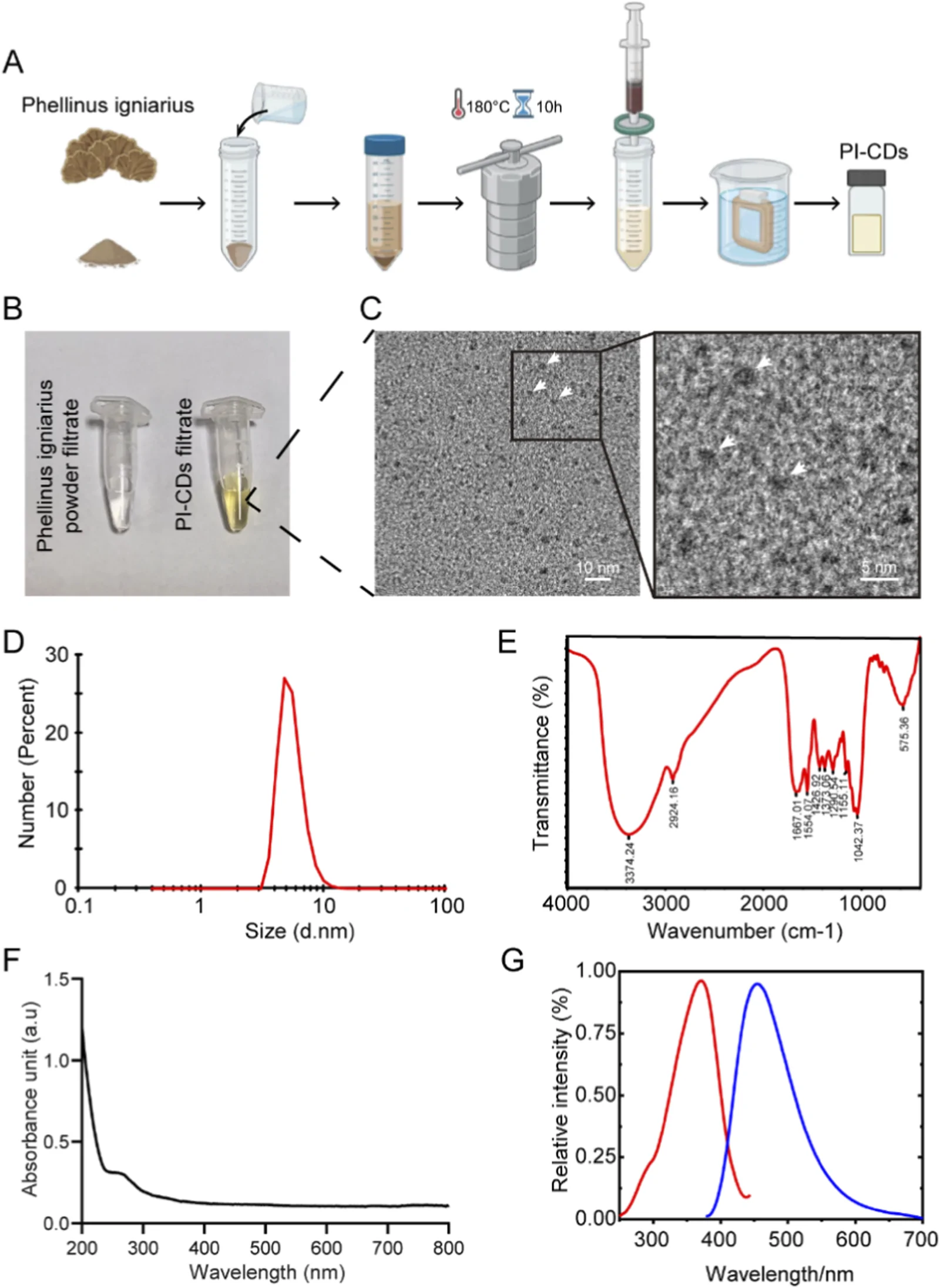
Characterization of PI-CDs. **(A)** Schematic illustration of the preparation process of PI-CDs. **(B)** Macroscopic photographs of Phellinus igniarius powder filtrate and PI-CDs filtrate. **(C)** TEM image of PI-CDs. **(D)** Hydrodynamic size distribution of PI-CDs determined by DLS. **(E)** FTIR spectrum of PI-CDs. **(F)** UV–vis absorption spectrum of PI-CDs. **(G)** Excitation and emission spectra of PI-CDs.

To confirm the successful synthesis and physicochemical properties of Phellinus igniarius-derived carbon dots (PI-CDs), their morphology and structural characteristics were systematically characterized. PI-CDs were synthesized from Phellinus igniarius powder through hydrothermal carbonization process followed by filtration and purification (Figure 1A). Macroscopic observation showed that the filtrate of PI solution became nearly colorless and transparent after filtration through a 0.22 μm membrane, whereas the PI-CDs filtrate retained a distinct yellow color (Figure 1B), suggesting the formation of water-dispersible nanoproducts after carbonization. Transmission electron microscopy (TEM) images revealed that PI-CDs exhibited quasi-spherical nanoparticle morphology with good dispersibility and no obvious aggregation (Figure 1C). Dynamic light scattering (DLS) analysis demonstrated a relatively narrow size distribution, with an average hydrodynamic diameter of 7.60 ± 1.14 nm (Figure 1D). Fourier transform infrared spectroscopy (FTIR) was further employed to characterize the surface functional groups of PI-CDs. A broad absorption peak at 3377.10 cm^-1^ was attributed to the stretching vibrations of O–H and/or N–H groups. The absorption peak observed at 1664.28 cm^-1^ was associated with C=O stretching vibration, while the characteristic peak at 1607.84 cm^-1^ corresponded to aromatic C=C skeletal vibrations, indicating the presence of sp^2^-conjugated carbon structures. In addition, the absorption peaks at 1384.46 cm^-1^ and 1288.27 cm^-1^ were likely related to the vibrations of C–N and C–O functional groups, respectively (Figure 1E). These results suggested that PI-CDs possessed abundant oxygen- and nitrogen-containing surface functional groups. Ultraviolet–visible (UV–vis) absorption spectroscopy showed that PI-CDs exhibited a strong absorption band in the ultraviolet region with a characteristic shoulder peak around 260 nm, which may be attributed to the π–π* transition of the carbon core structure, indicating typical optical absorption characteristics of carbon-based nanomaterials (Figure 1F). Fluorescence spectroscopy further demonstrated that the maximum excitation wavelength of PI-CDs was 368 nm, with a corresponding maximum emission wavelength at 545 nm (Figure 1G). Collectively, these results demonstrated that PI-CDs with uniform particle size, good dispersibility, and characteristic structural features of carbon dots were successfully synthesized in this study.

### PI-CDs suppress malignant behaviors of tumor cells

**FIGURE 2:**
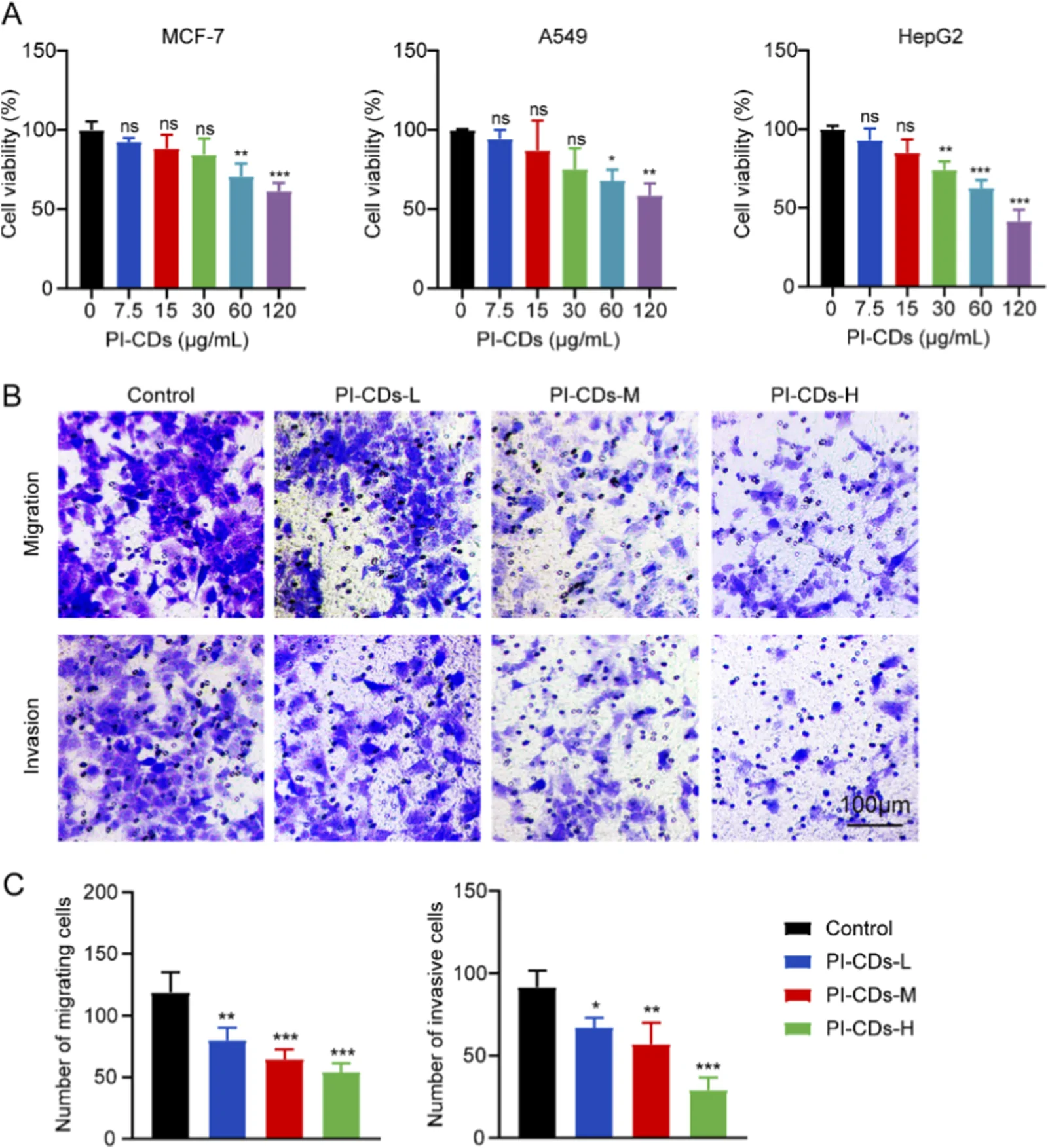
PI-CDs suppress malignant behaviors of tumor cells. **(A)** Effects of PI-CDs on tumor cell viability determined by CCK-8 assay. **(B)** Representative images of Transwell migration and invasion assay after PI-CDs treatment. **(C)** Quantitative analysis of cell migration and invasion. Data are presented as mean ± SD (n = 3). *P < 0.05, **P < 0.01, ***P < 0.001 versus control group; ns, not significant.

To evaluate the *in vitro* antitumor activity of PI-CDs, the effects of PI-CDs on the viability of different tumor cell lines were assessed using the CCK-8 assay. The results showed that PI-CDs exerted dose-dependent inhibitory effects on MCF-7, A549, and HepG2 cells. With increasing PI-CDs concentrations, cell viability gradually decreased across all tested cell lines (Figure 2A). At the highest tested concentration of 120 μg/mL, the cell viabilities of MCF-7, A549, and HepG2 cells were reduced to 61.52% ± 5.20%, 58.53% ± 7.70%, and 41.5% ± 7.45%, respectively. Notably, HepG2 cells exhibited greater sensitivity to PI-CDs compared with MCF-7 and A549 cells, as evidenced by a more pronounced reduction in cell viability under the same treatment conditions. Based on these findings, HepG2 cells were selected for subsequent mechanistic and functional studies. The effects of PI-CDs on the migratory and invasive capacities of HepG2 cells were further evaluated using Transwell assays. PI-CDs treatment significantly reduced both the number of migrated and invaded cells compared with the control group, and this inhibitory effect became more pronounced in a concentration-dependent manner (Figures 2B,C). At a concentration of 60 μg/mL, PI-CDs treatment reduced the migratory and invasive abilities of HepG2 cells by approximately 54.5% and 68.4%, respectively, compared with the control group. Collectively, these results demonstrate that PI-CDs effectively suppress tumor cell viability while impairing migratory and invasive abilities, indicating their potential antitumor activity *in vitro*.

### PI-CDs exert antitumor effects through MAPK-related signaling pathways

**FIGURE 3:**
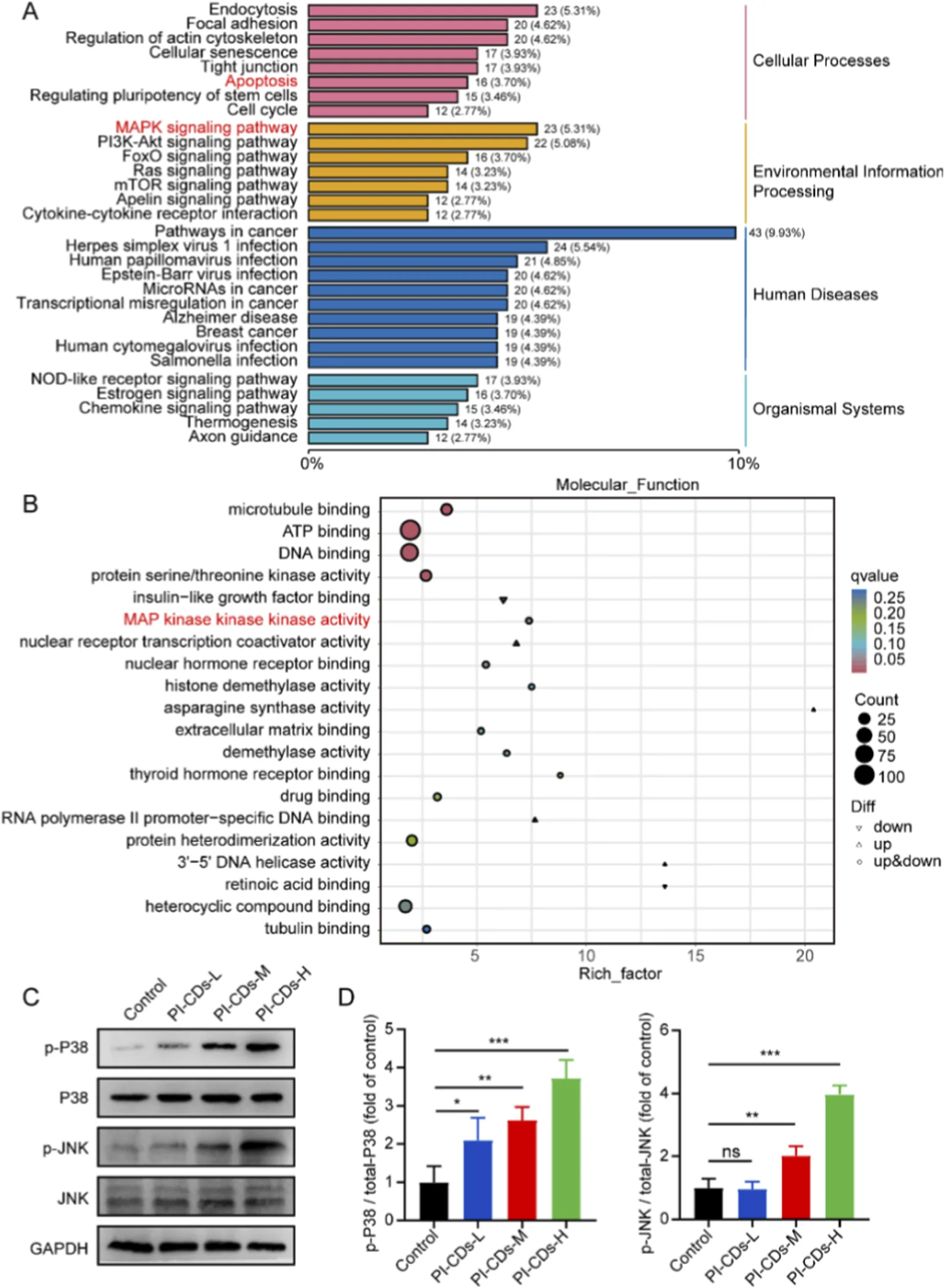
PI-CDs exert antitumor effects through MAPK-related signaling pathways. **(A)** KEGG pathway enrichment analysis of differentially expressed genes in PI-CDs-treated HepG2 cells. **(B)** GO enrichment analysis of differentially expressed genes. **(C)** Western blot analysis of total and phosphorylated p38 and JNK proteins in HepG2 cells following PI-CDs treatment. **(D)** Quantitative analysis of p-P38/P38 and p-JNK/JNK protein expression levels. Data are presented as mean ± SD (n = 3). *P < 0.05, **P < 0.01, ***P < 0.001 versus control group; ns, not significant.

To further investigate the molecular mechanisms underlying the inhibitory effects of PI-CDs on HepG2 cells, RNA-seq analysis and bioinformatic profiling were performed following PI-CDs treatment. KEGG pathway enrichment analysis revealed that differentially expressed genes were mainly enriched in several classical tumor-related pathways, including the MAPK signaling pathway, PI3K-Akt signaling pathway, FoxO signaling pathway, and apoptosis pathway (Figure 3A). Among these, the MAPK signaling pathway and apoptosis-related pathways showed relatively prominent enrichment, supporting the involvement of cellular stress responses and apoptosis-associated signaling alterations following PI-CDs treatment. Further Gene Ontology (GO) enrichment analysis demonstrated that the differentially expressed genes were mainly associated with molecular functions such as MAP kinase kinase kinase activity, DNA binding, and ATP binding (Figure 3B). Notably, GO analysis also identified kinase activity-related terms involved in MAPK cascade signaling, which was consistent with the KEGG enrichment results and suggested the potential involvement of MAPK signaling in the cellular response to PI-CDs treatment. To further validate the transcriptomic findings, the activation status of key MAPK signaling components was examined at the protein level. Western blot analysis demonstrated that PI-CDs treatment markedly increased the phosphorylation levels of P38 and JNK (Figures 3C,D). These results were consistent with the transcriptomic enrichment analysis and suggested that PI-CDs could trigger the activation of stress-responsive MAPK signaling pathways.

### PI-CDs induce ROS accumulation and promote mitochondria-dependent apoptosis

**FIGURE 4:**
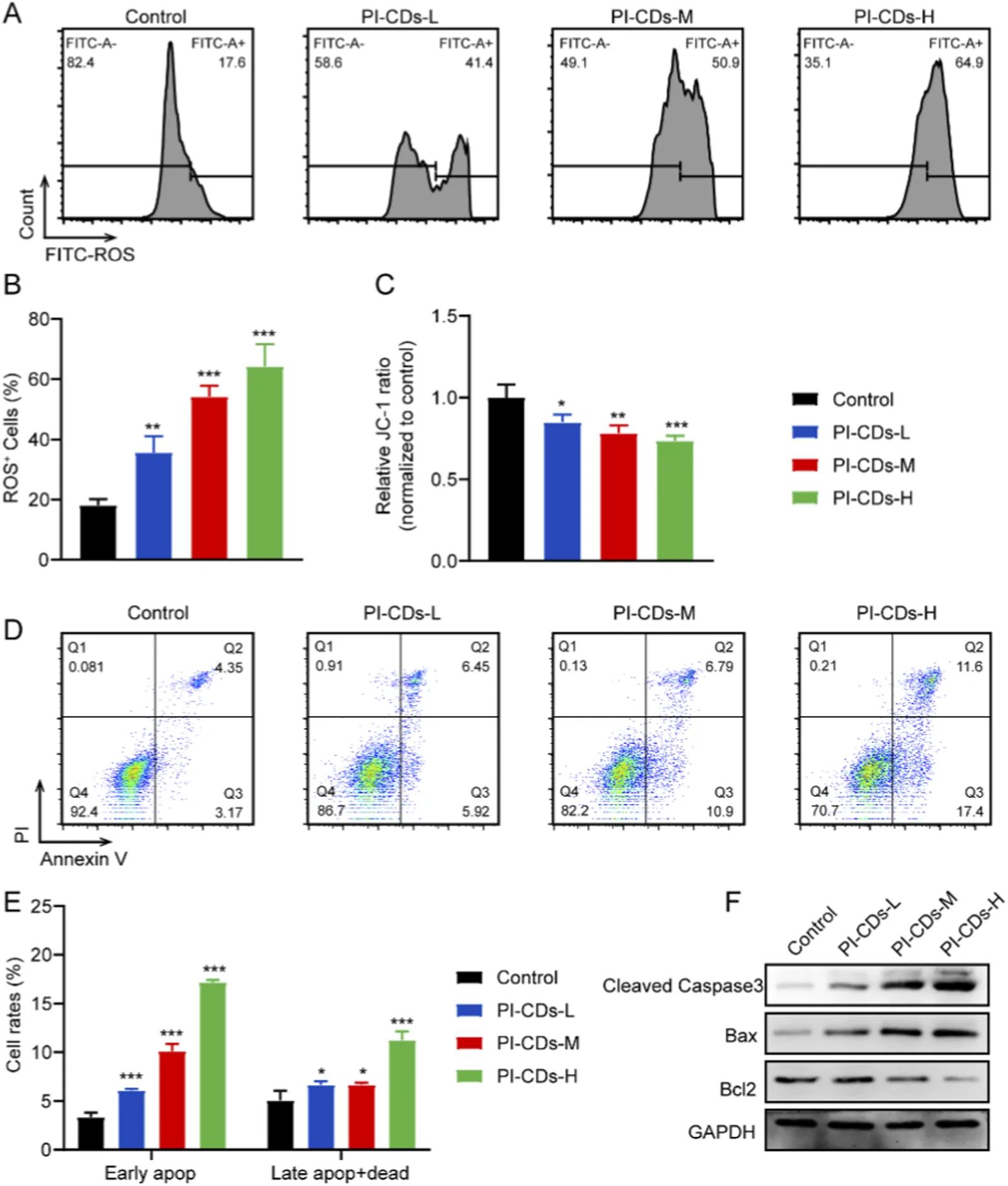
PI-CDs induce ROS accumulation and promote mitochondria-dependent apoptosis. **(A)** Flow cytometric analysis of intracellular ROS levels after PI-CDs treatment. **(B)** Quantitative analysis of ROS fluorescence intensity. **(C)** Detection of mitochondrial membrane potential using JC-1 staining after PI-CDs treatment. **(D)** Flow cytometric analysis of apoptosis using Annexin V-FITC/PI staining. **(E)** Quantitative analysis of apoptotic cell percentage. **(F)** Western blot analysis of apoptosis-related proteins in PI-CDs-treated HepG2 cells. Data are presented as mean ± SD (n = 3). *P < 0.05, **P < 0.01, ***P < 0.001 versus control group.

Accumulating evidence has demonstrated that MAPK signaling serves as a critical regulator of cellular stress responses and is highly responsive to alterations in intracellular redox homeostasis. In particular, activation of the p38 and JNK pathways is frequently associated with oxidative stress-mediated cellular events, including mitochondrial dysfunction and apoptosis. Therefore, the observed activation of MAPK signaling following PI-CDs treatment suggested the potential involvement of oxidative stress in the cellular responses induced by PI-CDs. To further investigate this possibility, intracellular reactive oxygen species (ROS) levels were evaluated in HepG2 cells following PI-CDs treatment. Flow cytometric analysis revealed that intracellular ROS levels were significantly elevated in PI-CDs-treated HepG2 cells compared with the control group, indicating that PI-CDs induced oxidative stress responses in tumor cells (Figures 4A,B). ROS are important intracellular signaling molecules that not only participate in oxidative stress responses but also regulate cell fate through modulation of mitochondrial function. Based on the close association between ROS accumulation and mitochondrial dysfunction, mitochondrial membrane potential was further assessed using JC-1 staining. PI-CDs treatment markedly reduced the red/green fluorescence intensity ratio, indicating mitochondrial membrane depolarization and impaired mitochondrial function (Figure 4C). Loss of mitochondrial membrane potential is widely recognized as an important hallmark of mitochondrial dysfunction and an early event during apoptosis. To further determine whether PI-CDs-induced mitochondrial dysfunction was associated with apoptosis, Annexin V-FITC/PI double staining was performed followed by flow cytometric analysis. The results demonstrated that PI-CDs treatment significantly increased the apoptotic rate of HepG2 cells in a concentration-dependent manner (Figures 4D,E). Furthermore, Western blot analysis revealed that PI-CDs treatment increased the expression of the apoptosis-promoting proteins cleaved caspase-3 and Bax, while decreasing the expression of the anti-apoptotic protein Bcl-2 in HepG2 cells (Figure 4F), further confirming the activation of the mitochondrial apoptotic pathway. Collectively, these findings indicate that PI-CDs induce intracellular ROS accumulation, accompanied by mitochondrial dysfunction and enhanced apoptosis in HepG2 cells. Combined with the activation of P38/JNK signaling observed in the preceding experiments, these results suggest that oxidative stress-associated MAPK signaling may related to the antitumor activity of PI-CDs.

### PI-CDs exhibit antitumor activity *in vivo*

**FIGURE 5:**
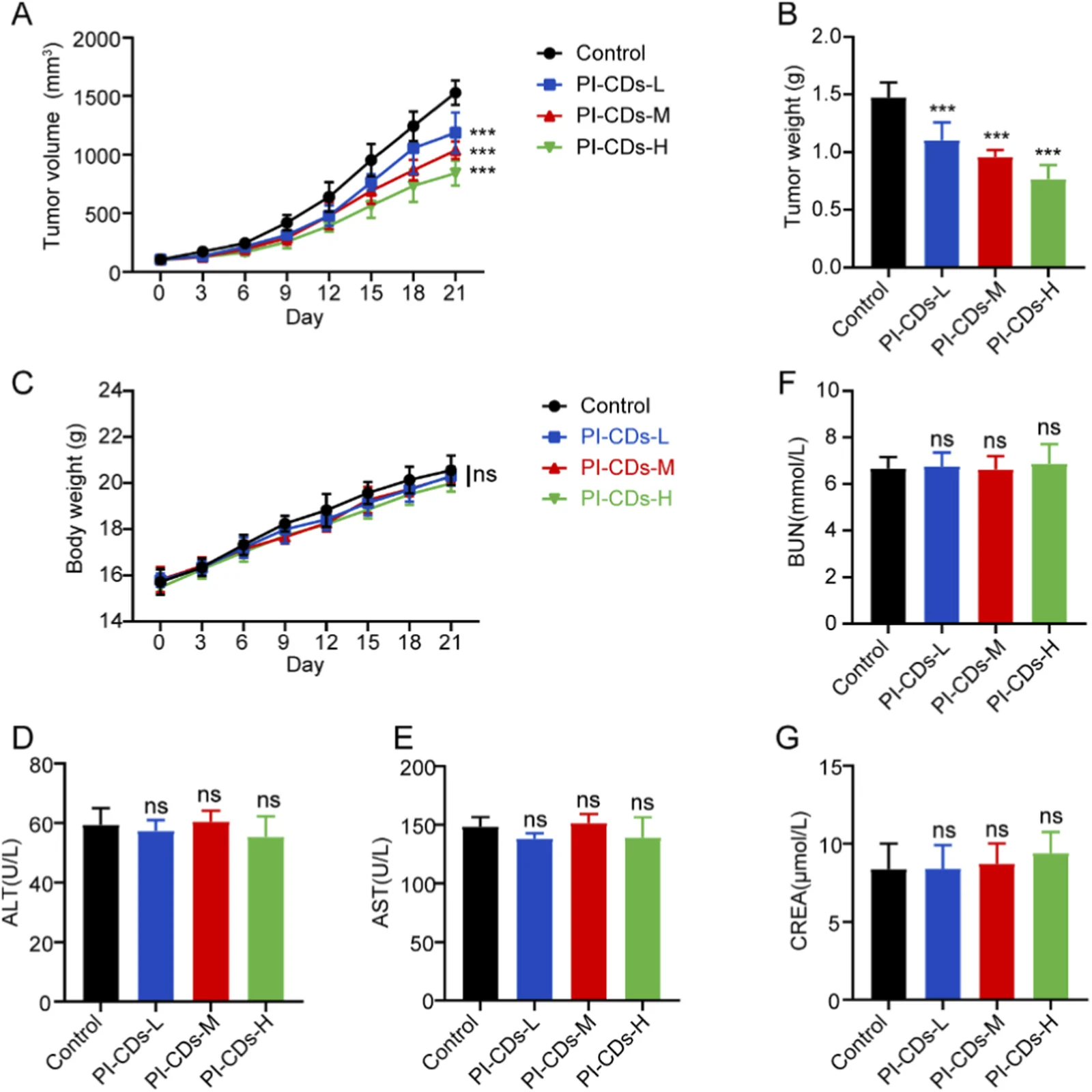
PI-CDs exhibit antitumor activity *in vivo*. **(A)** Tumor growth curves of HepG2 tumor-bearing mice following PI-CDs treatment. **(B)** Tumor weights of HepG2 tumor-bearing mice. **(C)** Body weight changes during treatment. **(D)** Serum alanine aminotransferase (ALT) levels. **(E)** Serum aspartate aminotransferase (AST) levels. **(F)** Blood urea nitrogen (BUN) levels. **(G)** Serum creatinine (CREA) levels. Data are presented as mean ± SD (n = 6). ***P < 0.001 versus control group; ns, not significant.

Based on the antitumor effects observed *in vitro*, a HepG2 xenograft tumor model was further established to evaluate the *in vivo* anontitumor activity of PI-CDs. The results demonstrated that PI-CDs treatment markedly suppressed tumor growth compared with the control group. Moreover, increasing doses of PI-CDs resulted in a greater inhibitory effect on tumor growth, as evidenced by the reduced tumor volume progression, indicating a dose-dependent antitumor effect (Figure 5A). At the experimental endpoint, tumor weights in all PI-CDs-treated groups were significantly lower than those in the control group (Figure 5B). In addition, no significant differences in body weight were observed between PI-CDs-treated groups and the control group throughout the treatment period (Figure 5C), indicating no apparent adverse effects on overall health status under the tested conditions. To further verify the biosafety of PI-CDs, serum biochemical analyses were performed in healthy mice after PI-CDs administration. The levels of alanine aminotransferase (ALT) and aspartate aminotransferase (AST), which are commonly used indicators of liver function, showed no significant differences between PI-CDs-treated groups and the control group (Figures 5D,E). Similarly, no significant changes were observed in blood urea nitrogen (BUN) and serum creatinine (CREA), indicators of renal function, following PI-CDs administration (Figures 5F,G). These results indicate that PI-CDs exhibited favorable preliminary systemic biocompatibility at the tested doses while maintaining potent *in vivo* antitumor activity.

## Discussion

Natural product-derived carbon dots (CDs) have emerged as a promising class of nanomaterials for cancer therapy due to their unique physicochemical properties, including nanoscale size, tunable surface functional groups, and high biocompatibility (Farshbaf et al., 2018; Gonzalez and Romero, 2025). These features enable CDs not only to serve as imaging probes or drug carriers but also to exert intrinsic bioactivity, such as modulation of oxidative stress and induction of cancer cell apoptosis (Yan et al., 2025; Chen et al., 2025). Despite growing interest, the therapeutic potential and underlying mechanisms of CDs derived from medicinal fungi remain largely unexplored. Our study demonstrates that PI-CDs, obtained from thermally processed Phellinus igniarius, can act as a bioactive nanomaterial with significant antitumor effects, providing new insight into the potential of medicinal fungi as sources of nanoscale therapeutics.

Reactive oxygen species (ROS) are critical regulators of cancer cell fate, functioning as signaling molecules at moderate levels while triggering oxidative damage and apoptosis when overproduced (Mohamed et al., 2025; Zhao et al., 2025). The nanoscale size and rich surface chemistry of CDs allow them to interact with intracellular redox systems, potentially perturbing ROS homeostasis. Accumulating evidence suggests that excessive ROS can disrupt mitochondrial membrane potential, promote cytochrome c release, and activate caspase cascades, ultimately leading to apoptosis. PI-CDs may leverage this property, as suggested by our findings, to selectively induce oxidative stress in cancer cells. Similar mechanisms have been reported for other natural product-derived CDs, including triterpenoid-derivesd CDs, supporting the concept that ROS-mediated mitochondrial dysfunction is a general pathway for CD-induced cytotoxicity (Tian et al., 2023).

The MAPK signaling pathway serves as a central hub linking ROS signaling to cellular stress responses, viability, and apoptosis. ROS accumulation can activate ERK, JNK, and p38 MAPK cascades, leading to either cell survival or apoptosis depending on the cellular context (Kwak et al., 2023; Zhang et al., 2025; Lee et al., 2025). In particular, activation of JNK and p38 MAPK is closely associated with mitochondrial outer membrane permeabilization and caspase activation (Jia et al., 2021; Nadeem et al., 2024). Our transcriptomic analyses indicate that PI-CDs treatment modulates multiple MAPK-related genes and apoptosis-related pathways, suggesting that ROS accumulation induced by PI-CDs may trigger downstream MAPK signaling to orchestrate cell death. These findings are consistent with previous reports linking natural nanomaterials, ROS modulation, and MAPK-mediated apoptosis in cancer cells (Su et al., 2019; Wu et al., 2022). Collectively, integration of cellular, biochemical, and transcriptomic data suggests that PI-CDs may exert antitumor effects through a coordinated regulatory network involving oxidative stress amplification and MAPK-mediated apoptotic signaling, ultimately leading to mitochondrial dysfunction and programmed cell death in liver cancer cells. Notably, transcriptomic analysis also revealed significant enrichment of other signaling pathways, including PI3K-Akt and FoxO signaling. Although these pathways were not further investigated in the present study, they have been implicated in the regulation of cell survival, metabolism, oxidative stress, and apoptosis (Shi et al., 2026; Li et al., 2026; Guo et al., 2022; Liu et al., 2018; Shukla et al., 2014), suggesting that they may also contribute to the biological effects of PI-CDs. Therefore, ROS-associated MAPK signaling and mitochondria-dependent apoptosis likely represent major mechanisms underlying the antitumor activity of PI-CDs rather than the only pathways involved.

In summary, our study highlights the potential of PI-CDs as a novel antitumor nanomaterial derived from a medicinal fungus. The underlying mechanism likely involves ROS-mediated mitochondrial dysfunction and activation of MAPK-related apoptotic pathways. These findings provide a framework for the rational design of bioactive CDs from thermally processed natural products and offer insights into the broader application of medicinal fungi in nanomedicine. Future work may focus on further elucidate the precise molecular mechanisms underlying PI-CDs’ antitumor effects, evaluate their efficacy across additional cancer models, and explore potential synergistic strategies with established chemotherapeutic agents.

## Data Availability

The data presented in the study are deposited in the NCBI Sequence Read Archive (SRA) repository, accession number PRJNA1518819.
